# miR-940 Suppresses Tumor Cell Invasion and Migration via Regulation of CXCR2 in Hepatocellular Carcinoma

**DOI:** 10.1155/2016/7618342

**Published:** 2016-10-11

**Authors:** Dong Ding, Yaodong Zhang, Renjie Yang, Xing Wang, Guwei Ji, Liqun Huo, Zicheng Shao, Xiangcheng Li

**Affiliations:** Liver Transplantation Center, First Affiliated Hospital of Nanjing Medical University, Key Laboratory of Living Donor Liver Transplantation, Ministry of Public Health, Nanjing, China

## Abstract

*Aim*. To investigate the expression of miR-940 in the hepatocellular carcinoma (HCC) and its impact on function and biological mechanism in the HCC cells.* Methods*. Quantitative RT-PCR analysis was used to quantify miR-940 expression in 46 cases of tissues and cells. Transfection of HCC cell lines was performed by miR-940 mimics; the abilities of invasion and migration were assessed through Transwell array. Western blot represents the alteration in expression of CXCR2 by miR-940 mimics.* Results*. miR-940 expression was decreased significantly in the HCC tissues and the relevant cell lines. miR-940 upregulation suppressed the invasion and migration of HCC cells in vitro. Furthermore, the CXCR2 was downregulated to suppress invasion and migration after miR-940 mimics. Moreover, decreased miR-940 expression was negatively correlated with Edmondson grade (*P* = 0.008), tumor microsatellite or multiple tumors (*P* = 0.04), vascular invasion (*P* = 0.035), and recurrence and metastasis (*P* = 0.038). Kaplan-Meier analysis demonstrated that decreased miR-940 expression contributed to poor overall survival (*P* < 0.05).* Conclusions*. Our findings present that miR-940 acts as a pivotal adaptor of CXCR2 and its transcription downregulated CXCR2 expression to decrease HCC invasion and migration in vitro. Our study suggests that miR-940 may be a novel poor prognostic biomarker for HCC.

## 1. Introduction

Hepatocellular carcinoma (HCC) accounts for up to 90% of all primary liver cancers worldwide and is one of the most common malignant tumor in China [1]. Despite advances we have achieved in surgery therapy approaches, the overall prognosis of HCC is still far from satisfactory due to high incidences of tumor recurrence and metastasis. MicroRNA (miRNAs) are a recently discovered class of endogenous, small (about 20–24 nucleotides), noncoding RNAs, which play important roles in cancer development and progression [2–4]. By posttranscriptional regulation of target gene, miRNAs are involved in diverse processes such as cell proliferation, differentiate, apoptosis, and metabolism [5]. Currently, miRNAs are associated with occurrence and progression of many diseases including the malignancy tumors.

Recently, several studies have revealed the role of miR-940 in various types of cancer. We have found that miR-940 were downregulated in many malignant tumors such as prostate cancer and pancreatic carcinoma, and it is also relevant to tumor invasion and metastasis. Therefore, miR-940 may be a novel poor prognostic biomarker of hepatocellular carcinoma.

The recent studies show that inflammation is a recognized risk factor in cancer development and progression [6]. Many inflammatory cytokines and chemokines contribute to cancer invasion and metastases [7]. Chemokines are small (8–14 kDa) chemoattractant cytokines that selectively regulate the recruitment and trafficking of leukocyte subsets to sites of inflammation. Chemokines have been divided into four subfamilies: CC, CXC, XC, and CX3C. CXCR2 is a member of the G-protein-coupled receptor superfamily and the receptor of CXC chemokines: CXCL1, CXCL2, CXCL3, CXCL5, and CXCL7 bind specifically to CXCR2; CXCL6 and CXCL8 (interleukin 8, IL8) are shared ligands of CXCR1 and CXCR2. Recently, studies demonstrated that CXCR2 play a critical role in tumor invasion and metastases in many types of cancer, such as lung cancer, melanoma, colon cancer, and pancreatic cancer [8–12]. So far the tie between miR-940 and CXCR2 was unclear in the HCC.

To investigate the role of miR-940 in the malignant behavior of HCC, our data indicated that lower expression of miR-940 was evaluated to have an effect on the migration and invasion of HCC in vitro; we also showed that miR-940 overexpression suppressed cell migration and invasion and transcription of CXCR2 and thereby inhibit invasion and migration of HCC. Moreover, patients with lower levels of miR-940 expression had a relatively poor biological characteristics and prognosis. Our findings provide insight that the further prospective value of miR-940 may be a novel poor prognostic biomarker for HCC.

## 2. Materials and Methods

### 2.1. Clinical Samples and Histology

Tumor specimens and matched tumor-adjacent normal tissues were obtained from 46 HCC patients without preoperative systemic chemotherapy, who underwent therapeutic removal of hepatocellular carcinoma at The First Affiliate Hospital of Nanjing Medical University. All HCC tissues were collected using protocols approved by the Ethics Committee of The First Affiliate Hospital of Nanjing Medical University, and written informed consent was obtained from every patient. All of the patients were diagnosed as primary hepatocellular carcinoma based on pathological evidence. Tissue samples were obtained and immediately frozen in liquid nitrogen. Samples were stored at −80°C until use.

### 2.2. Cell Culture and Transfection

All cell lines were obtained from the Cell Bank of the Chinese Academy of Science (Shanghai, China). Cells were maintained in DMEM medium (Gibco USA) supplemented with 10% fetal bovine serum (FBS) and 10 lg/mL fibronectin (BD Biosciences) and penicillin/streptomycin 100 units/mL at 37°C in a humidified incubator containing 5% CO_2_. The control and miR-940 mimics were obtained from Genechem Biotechnologies Co., Ltd. The targeting sequences in miR-940 were 5′-AAGGCAGGGCCCCCGCUCCCC-3′, 5′-GGAGCGGGGGCCCUGCCUUUU-3′. The control target sequences were 5′-CAGUACUUUUGUGUAGUACAA-3′. All of the transfection assays were performed with Lipofectamine 2000 transfection reagent (Invitrogen, Carlsbad, CA) in accordance with manufacturer's protocol.

### 2.3. Cell Migration and Invasion Assay

HCC cell lines, MHCC97H and SMMC-7721 (1 × 10^5^ cells/well), in serum-free media were seeded in the upper Transwell chambers (BD Biosciences) with complete media as a chemoattractant in the lower chamber. After a 48 h incubation period at 37°C, 5% CO_2_, the media and cells remaining in the upper chamber were removed using a cotton bud. The insert was fixed in ethanol and stained with crystal violet. The number of the cells on the lower surface was counted by microscope.

The invasion assay was performed in the same manner as the migration assay except that the upper chamber was precoated with 50 g/well of Matrigel overnight before seeding cells.

### 2.4. RNA Preparation and qRT-PCR

Total RNAs were extracted from tumor and adjacent normal tissues or cultured cells using TRIzol solution (Invitrogen) following the manufacturer's protocol. The miR-940 expressions of HCC, adjacent tumor issues, HeP3B, MHCC-97L, SMMC-7721, HL-7702 were tested by qRT-PCR. The A260/A280 ratio was used to evaluate RNA purity. Quantitative real-time PCR was performed by the Thermal Cycler Dice Detection System with the SYBR Premix Ex TaqTM (Takara). The forward primers used in this study is 5′-CCTGTCTTACTTTTCCGAAGGAC-3′, and reverse primer is 5′-TTGCTGTATTGTTGCCCATGT-3′. The qRT-PCR was performed in triplicate, and the relative expression of miR-940 was calculated using the comparative cycle threshold (CT) (2^−ΔΔCT^) method with u6 rRNA as the endogenous control to normalize the data and relative quantitative expression of miR-940 was calculated to identified.

### 2.5. Western Blot

Cells and tissue samples were lysed using RIPA protein extraction reagent. Proteins were separated on 8% sodiumdodecylsulfate-polyacrylamide gel electrophoresis (SDS-PAGE), transferred to nitrocellulose membranes (Sigma) and incubated in 4°C for one night with appropriate antibodies. The membranes were incubated about 2 h with goat anti-rabbit IgG antibody. The bands were visualized by Bio-Rad. The comparative expressions of target stripes were estimated through the comparison of B-actin stripes. Antibodies (1 : 1000 dilutions) against CXCR2 were purchased from Cell Signaling Technology. Optimal exposure times of membranes were used, and protein expression was quantified with the use of the NIH Image J (National Institutes of Health, Bethesda, MD) and adjusted for background noise and protein loading.

### 2.6. Statistics

All statistical analyses were performed using SPSS 18.0 software (IBM, SPSS, Chicago, IL, USA). The significance of the differences between groups was estimated by the Student's *t*-test, *χ* ^2^ test as appropriate. Two-sided *P* values were calculated, and a probability level of 0.05 was chosen for statistical significance.

## 3. Results

### 3.1. miR-940 Was Downregulated in Tissues and Cell Lines

**Figure 1:**
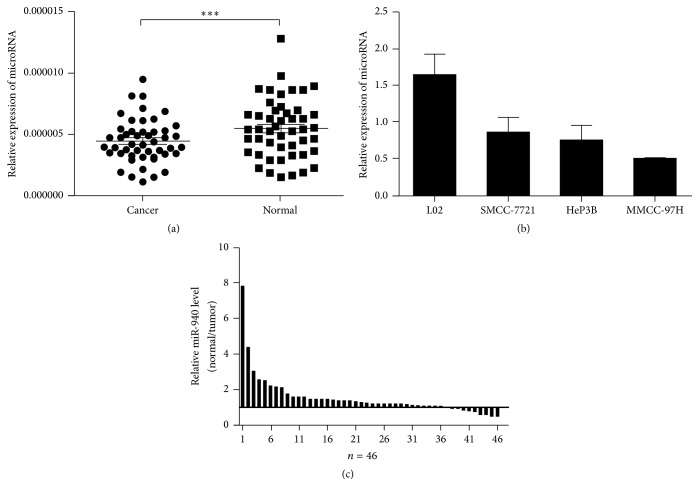
Downregulation of miR-940 in HCC tissues and cell lines. (a) The miR-940 expression in 46 pairs of HCC and adjacent normal tissues (non-HCC). (b) The expression of miR-940 in three HCC cell lines with different metastatic potentials as well as the normal human liver cell line L02. (c) Relative expression of miR-940 in HCC tissues and adjacent normal tissues. All the experiments were iterated at least three times and the data are presented as mean with standard deviation. ^*∗∗∗*^ *P* < 0.001 versus the control. U6rRNA was used as an internal control.

MiR-940 expression levels were investigated in 46 couples of hepatocellular carcinoma samples and adjacent normal tissues using qRT-PCR assays. Compared with the adjacent normal tissues, the expressions of HCC were examined to be significantly lower level (*P* < 0.05Figure 1(a)). The qRT-PCR assays were further developed to quantify miR-940 in hepatocellular carcinoma cell lines. The outcomes revealed that the expression levels of miRNA in MHCC-97H and SMMC-7721 were less than normal human liver cells HL-7702 (*P* < 0.05Figure 1(b)).

### 3.2. miR-940 Expression and Clinicopathological Factors in Hepatocellular Carcinoma

To assess the correlation of miR-940 expression with clinicopathological data, miR-940 expression levels in tumor tissues were categorized as low or high in relation to the median value of relative miR-940 expression. Clinicopathological factors were analyzed in the high and low miR-940 expression groups. As shown in Table 1, the low miR-940 group showed higher Edmondson grade, tumor microsatellite or multiple tumors, and advanced vascular invasion. However, there was no significant correlation between miR-940 expression and other clinicopathological features, such as age, gender, etiology, cirrhosis, AFP, tumor size, and TNM stage.

### 3.3. miR-940 Exhibits Significant Effect on HCC Invasion and Metastasis

**Figure 2:**
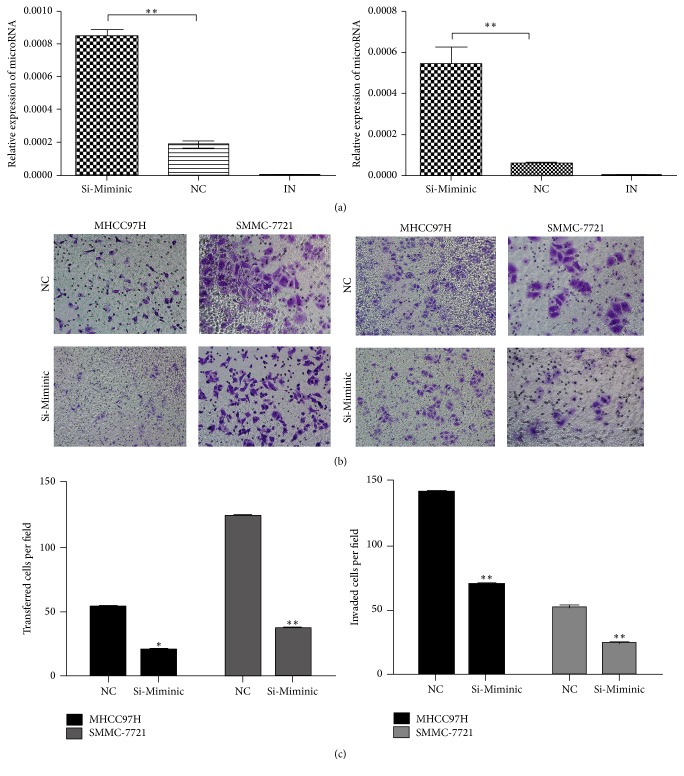
Overexpression of miR-940 promotes HCC cell migration and invasion in vitro. (a) Upregulation of miR-940 in MHCC97H and SMMC-7721 was demonstrated by qRT-PCR. (b) Transwell assay to observe the effect of miR-940 overexpression on cell migration capacity. (c) Cell invasion assay to assess the effect of miR-940 overexpression on cell invasion capacity. The histograms represent mean with standard deviation of the number of invasive or migration cells from triplicate tests. All experiments were performed independently three times. Student's *t*-test was used to evaluate the statistical significance of these experiments, as compared to NC. ^*∗*^ *P* < 0.05, ^*∗∗*^ *P* < 0.01 versus the control. Original magnification, ×100.

The significant low expression of miR-940 in HCC samples prompted us to explore its possible biologic significance in tumorigenesis. The overexpression of miR-940 in cells was confirmed by qRT-PCR. The analysis suggested that the migration and invasion of MHCC97H and SMMC-7721 were suppressed significantly with miRNA transfection (Figure 2).

The interaction prediction by TargetScan and miRanda suggests the interaction between CXCR2 and miR-940 in the HCC cells.

### 3.4. Low miR-940 Expression Is Associated with Poor Prognosis of Patients with Hepatocellular Carcinoma

Kaplan-Meier analysis and log-rank test were used to evaluate the effects of LINC00982 expression and the clinicopathological characteristics on 3 years' overall survival (OS). The results showed that patients in the low miR-940 expression group had shorter overall survival (median OS 19.7 months) than those in the high miR-940 expression group (median OS 29.1 months; *P* < 0.05Figure 4).

### 3.5. miR-940 Overexpression Reduced CXCR2 Expression in HCC Cells

**Figure 3:**
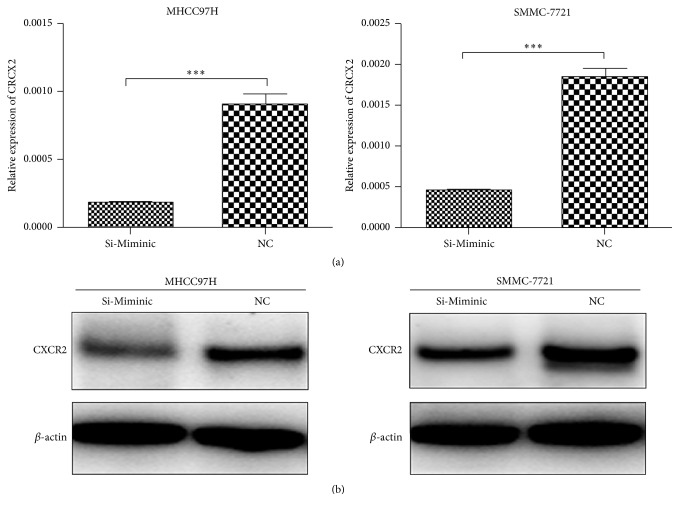
Overexpression of miR-940 reduced CXCR2 expression in vitro. (a) The expression mRNA level of CXCR2 in MHCC97H and SMMC-7721 was examined by qRT-PCR. (b) Expression of CXCR2 in MHCC- 97H and MHCC-LM3 demonstrated by Western blot assay, with *β*-actin as a loading control. ^*∗∗∗*^ *P* < 0.001.

**Figure 4:**
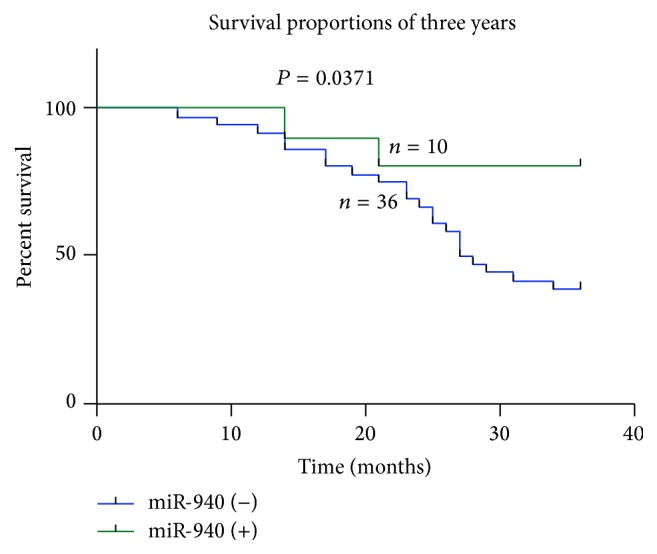
Kaplan-Meier overall survival curves of HCC patients according to the level of miR-940 expression. Overall survival of patients with HCC based on miR-940 expression status (*P* < 0.05).

In order to study the pathological significance of miR-940 downregulation in hepatocellular carcinoma cell lines, MHCC97H and SMMC-7721 cells were transfected with either control or mimics for miR-940 (Figure 3(a)). Western blot analysis revealed that miR-940 overexpression reduced CXCR2 expression (Figure 3(b)). The expression mRNA level of CXCR2 was examined by qRT-PCR. miR-940 overexpression downregulated CXCR2 expression by 5-fold compared with mock-transfected cells. These results showed that miR-940 overexpression downregulates CXCR2 expression.

## 4. Discussion

miRNA plays important roles in the regulation of gene expression. miRNAs are thought to activate the suppress function on the target gene by the complementary pairing between the sequence of target mRNA and the subsequences completely or in part [13]. There are many studies showing that miRNA is the important part of tumorigenesis; it has been demonstrated that many kinds of miRNAs have the function in cancer promotion or cancer suppression [14–16]. At present, the studies have proved that miR-940 was significantly downregulated in malignant tumors, such as gastric cancer and prostate cancer, through the regulation of malignant tumor apoptosis and the suppression of malignant tumor migration. Our results proved that obviously miR-940 is downexpressed in the liver tumor cells and tissues. Furthermore, we demonstrated for the first time that low miR-940 expression is associated with tumor cells invasion and migration [17–19].

Thereby, to further determine the clinical significance of miR-940, we inferred that miR-940 was correlated with the invasion and migration of hepatocellular carcinoma cells. The results revealed that low miR-940 expression was more frequently detected in tumors with higher Edmondson grade, more tumor microsatellite or multiple tumors, advanced vascular invasion, and more frequent recurrence and metastasis. Kaplan-Meier analyses were performed to confirm the prognostic value of miR-940. Our results showed that patients with a lower expression of miR-940 seemed to have shorter overall than patients with higher levels. According to the clinicopathological features, Edmondson grade, tumor microsatellite or multiple tumors, vascular invasion and recurrence, and metastasis, of HCC patients, we think that these factors may be playing pivotal role in the influence of poor prognosis. miR-940 might serve to identify high-risk individual patients with HCC who have higher risk of death and, thus, are good candidates for receiving more aggressive treatment. Meanwhile, limitations should also be noted that the number of specimens in this study is limited, and we need more cases to draw a more objective conclusion. The invasion and migration of malignant tumors were activated by many molecule pathways, and these regulatory molecules are the key roles in promoting the malignant tumor metastasis [17].

The contribution of inflammation in carcinogenesis is well appreciated. Inflammatory mediators are known to play crucial roles in tumor progression by regulating not just the immune infiltrations in tumors but also by playing the important role in invasion and metastasis [20, 21]. A number of studies have demonstrated that CXCR2 plays a pivotal role in tumor angiogenesis, proliferation, and invasion [8, 22, 23]. Up to now, however, the relationship between CXCR2 and miR-940 in HCC has been little noticed. In this study, we investigated the role of CXCR2 in the invasion and metastases using in vitro approaches.

After overexpression of miR-940, the CXCR2 expression of HCC cells is downregulated; this fact showed that the suppression of CXCR2 in the migration and invasion may be regulated by miR-940. In conclusion, our study for the first time showed that the migration and invasion may be significantly suppressed by miR-940 transfection though the regulation of CXCR2 expression [24].

In conclusion, our data indicated that miR-940 negatively regulates cell invasion and migration in hepatocellular carcinoma though regulation of CXCR2, which results in promotion of invasion and metastasis. These findings further the understanding of HCC pathogenesis and progression and facilitate comprehension of the miR-940 directed diagnostics and therapeutics against this deadly disease.

## Figures and Tables

**Table 1:** Correlation between miR-940 and clinicopathological characteristics in 46 HCCs.

HCC parameters	Number of patients	miR-940 (−)	miR-940 (+)	*χ* ^2^	*P*
Gender					
Male	38	31	7	0.515	0.473
Female	8	5	3		
Age					
≥50 years old	36	29	7	0.080	0.777
<50 years old	10	7	3		
Etiology					
HBV+	38	30	8	0.000	1.000
HBV−	8	6	2		
Cirrhosis					
Positive	37	31	6	1.934	0.164
Negative	9	5	4		
AFP (ng/mL)					
≥400	29	22	7	0.021	0.885
<400	17	14	3		
Tumor size					
≥5 cm	26	21	5	0.012	0.913
<5 cm	20	15	5		
TNM stage					
I-II	25	18	7	0.584	0.445
III-IV	21	18	3		
Edmondson grade					
I-II	22	13	9	7.076	0.008^*∗∗*^
III-IV	24	23	1		
Tumor microsatellite or multiple tumors					
Yes	20	19	1	4.217	0.040^*∗*^
No	26	17	9		
Vascular invasion					
Yes	15	15	0	4.432	0.035^*∗*^
No	31	21	10		
Recurrence and metastasis					
Yes	29	26	3	4.313	0.038^*∗*^
No	17	10	7		

miR-940 (−) indicated that miR-940 expression was downregulated in tumor tissue as compared with its adjacent nontumor tissue; miR-940 (+) indicated no downregulation of miR-940 between tumor tissue and adjacent nontumor tissue. ^*∗*^ *P* < 0.05, ^*∗∗*^ *P* < 0.01.
